# Identifying Distinct Antibiotic Behavioural Profiles in Singapore’s General Population: A Latent Class Analysis

**DOI:** 10.3390/antibiotics15070671

**Published:** 2026-07-09

**Authors:** Huiling Guo, Angela Chow

**Affiliations:** 1 Department of Epidemiology and Preventive Medicine, Tan Tock Seng Hospital, Singapore 308433, Singapore; huiling.guo@nhghealth.com.sg; 2 Saw Swee Hock School of Public Health, National University of Singapore, Singapore 117549, Singapore; 3 Lee Kong Chian School of Medicine, Nanyang Technological University, Singapore 308232, Singapore

**Keywords:** antimicrobial resistance, antibiotic behavioural profiles, general population, Singapore, latent class analysis

## Abstract

Background: Inappropriate antibiotic use for upper respiratory tract infections (URTIs) is common. However, existing research typically examines single indicators or broad categories of misuse practices, without considering the probabilistic and co-occurring nature of varying behaviours. This study aims to identify and characterise distinct antibiotic behavioural profiles within a general population to inform personalised interventions. Methods: This is a broadly representative population-based study of adult Singapore residents between November 2020 and January 2021. Latent class analysis was first performed to identify distinct profiles, followed by multinomial logistic regression to determine individual characteristics associated with each profile, with interaction effects examined. Results: Amongst 2004 respondents, the majority were “antibiotic appropriates” (53.0%), followed by “antibiotic avoiders” (24.9%), and “antibiotic seekers” (22.2%). “Antibiotic seekers” expected antibiotics for cold/flu, hopped between doctors to source antibiotics, used leftover antibiotics, stopped antibiotic courses prematurely and perceived antibiotics as harmless and useful for treating cold/flu. Individuals with poor knowledge of antibiotic use (AOR 3.71, 95% CI 2.89–4.76, *p* < 0.001) and antimicrobial resistance (AOR 3.51, 95% CI 1.05–11.76, *p* = 0.042), low eHealth literacy (AOR 1.34, 95% CI 1.02–1.77, *p* = 0.038), and high trust in doctors (AOR 2.14, 95% CI 1.44–3.17, *p* < 0.001) were more likely to be “antibiotic seekers”. Older adults with lower education levels were particularly likely to be “antibiotic seekers”. Conclusions: Approximately one-in-five Singapore residents are antibiotic seekers. Targeted education during multiple clinic visits, given the high trust in doctors, can address antibiotic knowledge gaps and misperceptions, reducing antibiotic misuse.

## 1. Introduction

The silent pandemic of antimicrobial resistance (AMR) continues to manifest globally, with AMR-associated deaths already reaching half of the 10 million annual deaths projected by 2050 [1,2]. Inappropriate antibiotic use remains a key driver of AMR [3], particularly in the management of upper respiratory tract infections (URTIs) in the outpatient setting [4].

When making antibiotic decisions for URTI episodes, the lay public adopts a complex six-stage cycle [5], which includes recognising URTI treatment needs, gathering information from health and social sources, evaluating alternative treatments, assessing antibiotic accessibility, weighing consumption risks and benefits, and appraising utility of antibiotics for future URTIs, before repeating with subsequent episodes. Individuals vary across these stages, generating distinct behavioural profiles [6].

However, current assessments of poor antibiotic practices amongst the general public are over-simplified. They often rely on limited indicators such as obtaining or self-medicating with antibiotics from non-prescribed sources and failure to complete antibiotic courses [7], assuming that such indicators alone would sufficiently account for misuse, ignoring other potential measures. Furthermore, broad categorical classifications are typically used, treating individuals as either engaging in specific inappropriate antibiotic behaviours or not, thus overlooking the probabilistic and co-occurring nature of these behaviours. As a result, these approaches would inevitably fail to capture the complexity of antibiotic use behaviours amongst the general public, leading to design of ineffective one-size-fits-all interventions, as seen in mass campaigns [8]. Comprehensive behavioural profiling is therefore critical in the development of more tailored intervention strategies within the community to improve antibiotic use practices.

To date, extensive research has identified determinants of inappropriate antibiotic use [9], with studies examining how demographics, knowledge, health information seeking behaviours, and trust in doctors interplay with one another in driving misuse [10,11,12]. Yet, these investigations have relied on the same broad characterisations of self-medication, unwarranted use and non-adherence to prescribed courses [9,10,11,12]. Adopting this more nuanced perspective, this study hence aims to examine how established determinants relate to distinct behavioural profiles derived from the six-stage antibiotic decision-making cycle, with further explorations of attitudes towards AMR and communication channels to inform future education efforts.

## 2. Materials and Methods

### 2.1. Study Design and Study Population

This is a population-based survey conducted between November 2020 and January 2021 on a broadly representative sample of Singapore citizens and permanent residents aged 21 years and above, invited through a sampling frame described in a previous study [10].

### 2.2. Variable Definition and Measurement

Seventeen items were mapped against the categories defined by Duan et al. [5] into (1) need recognition, (2) information seeking, (3) alternative evaluation, (4) antibiotic obtaining, (5) antibiotic consumption and (6) post-consumption evaluation, to measure antibiotic use behaviours (Supplementary Table S1). To minimise the potential influence of the COVID-19 pandemic on responses, items pertaining to need recognition and alternative evaluation were explicitly framed with reference to the period before the COVID-19 pandemic. Five-point Likert scale responses (1—Strongly disagree to 5—Strongly agree) were recoded as Strongly agree/Agree = 1 and Strongly disagree/Disagree/Neither agree nor disagree = 0, with reverse coding for negatively worded items. For the item, i.e., “Do you think that these conditions can be treated with antibiotics?—Common cold and flu”, with Yes/No/Don’t know responses (1-Yes, 0-No, 99-Don’t know), ‘Don’t know’ responses were recoded as No.

Knowledge of antibiotic use (3 items) and AMR (8 items) were measured using questions adapted from the World Health Organization’s Antibiotic Resistance Multi-country Public Awareness Survey [13], as previously reported [10]. Respondents were classified as having poor knowledge of antibiotic use and poor knowledge of AMR respectively, if they answered any question incorrectly.

eHealth literacy was measured using 7 binary items (1-Yes, 0-No) [14,15], as previously described [11]. An eHealth literacy score was tabulated using ‘Yes’ responses, and respondents were defined as having high eHealth literacy if they scored 7 out of 7 (75th percentile and above) and low eHealth literacy if they scored below 7 (below 75th percentile).

Overall trust in doctors was measured using 9 items presented on a 5-point Likert scale (1—Strongly disagree to 5—Strongly agree) adapted from Hall et al. [16], as previously reported [12]. Responses were recoded as 1 for Strongly agree/Agree responses on the 7 positively worded items and Strongly disagree/Disagree responses on the 2 negatively worded items, and 0 otherwise. These recoded values were summed to generate an overall trust in doctors score, and respondents were classified as having high overall trust in doctors if they scored 7 out of 9 (75th percentile and above) and low overall trust in doctors if they scored below 7 (below 75th percentile).

Attitudes towards antibiotic resistance were assessed through statements adapted from the World Health Organization’s Antibiotic Resistance Multi-country Public Awareness Survey [13], and participants were asked to rate their perceived effectiveness of public communication channels for AMR education methods. Both were presented on a 5-point Likert scale.

Basic demographic data, including age, ethnicity, and education level, and previous use of antibiotics were collected for all participants.

### 2.3. Latent Class Analysis

Latent class analysis (LCA) was conducted to classify the respondents into distinct subgroups using the 17 antibiotic use behaviour items mentioned above (Supplementary Table S1). The exact number of latent classes to be generated from the LCA model was determined based on the Akaike Information Criterion (AIC) and Bayesian Information Criterion (BIC), with lower criteria values indicating a better model fit (Supplementary Table S2). Class characteristics were evaluated using predicted class probabilities and class-specific item response probabilities (Supplementary Table S3). Individual respondents were assigned to their most likely class based on maximum posterior probability. All LCA analyses were conducted in Stata version 18.0 (StataCorp LLC, College Station, TX, USA).

### 2.4. Multinomial Logistic Regression

Categorical variables were summarised using proportions and continuous variables using means. Chi-squared tests and one-way ANOVA were used to test for significance between distinct classes, respectively. Multinomial logistic regression was performed to identify sociodemographic and other characteristics that predict membership into each class, with the largest class as the reference category. Known confounders (age, gender and ethnicity) were included a priori in all models, with other covariates selected based on likelihood ratio tests, and AIC/BIC values (Supplementary Table S4). Interactions between covariates were individually explored, and only interaction terms that meaningfully improved model fit, as assessed by likelihood ratio tests and AIC/BIC comparisons, were retained in the final regression model. Statistical significance was defined as a *p*-value < 0.05. Multinomial logistic regression analyses were also conducted in Stata version 18.0 (StataCorp LLC, College Station, TX, USA).

## 3. Results

### 3.1. Basic Characteristics of Respondents

Data were collected from 2004 Singapore residents, at a response rate of 41.8%. As reported previously [10], the sample was broadly representative across most demographic characteristics, including residency status, gender, ethnicity, housing type and marital status, although the study population slightly overrepresented adults younger than 50 years and those with higher education relative to the Singapore Census 2020. Respondent characteristics relevant to this study are presented in Table 1. The majority of the respondents had ever used antibiotics before (97.2%), had low eHealth literacy (67.5%) and low overall trust in doctors (65.9%). Even though more than half of the respondents had good knowledge of antibiotic use (59.3%), almost all of them had poor knowledge of AMR (97.0%).

### 3.2. Latent Class Profiles for Antibiotic Use Behaviours

**Figure 1:**
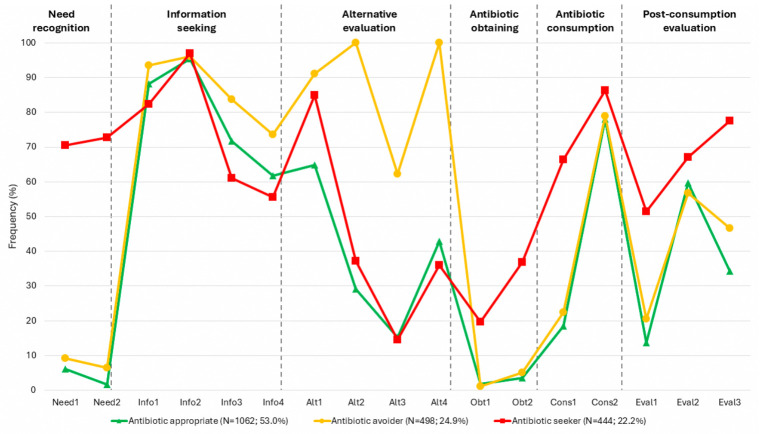
Frequency of items represented across latent class profiles for antibiotic use behaviours.

The respondents were classified into three main profiles: (1) Antibiotic appropriates (N = 1062; 53.0%), (2) Antibiotic avoiders (N = 498; 24.9%) and (3) Antibiotic seekers (N = 444; 22.2%) (Table 2). All three profiles did not differ in their ability to seek doctor’s advice or assistance for health-related matters (Figure 1).

“Antibiotic appropriates” were least likely to expect antibiotics for common cold and flu symptoms (6.1%) and to take antibiotics to prevent the common cold and flu from worsening (1.6%). They were also least likely to take leftover antibiotics based on personal judgement (3.5%), stop antibiotic courses prematurely (18.5%), perceive absence of harm from taking antibiotics (13.6%) and perceive usefulness of antibiotics to treat common cold and flu (34.3%). Nearly two-thirds of them (64.9%) consult a doctor to manage their symptoms when unwell.

“Antibiotic avoiders” were most likely to consult a doctor to manage their symptoms (91.2%) when unwell and least likely to see another doctor if not prescribed antibiotics (1.2%). In addition, they would always use (100.0%) over-the-counter Western medicine and rest to manage their symptoms. Six in ten of them (62.3%) would also use complementary and alternative medicine. Of interest, “antibiotic avoiders” displayed strong information seeking behaviours by being most likely to know where to seek health information (93.6%) and having family members (83.7%) and friends (73.7%) to discuss health issues with.

As compared to the other two profiles, “antibiotic seekers” had the highest proportion who would expect antibiotics for common cold and flu symptoms (70.5%) and to take antibiotics to prevent common cold and flu from worsening (72.8%). They were least likely to rest and let their body recover when unwell (36.0%), and most likely to doctor hop to get antibiotics (19.6%), use leftover antibiotics based on personal judgement (36.9%), stop antibiotic courses prematurely (66.4%), perceive absence of harm from taking antibiotics (51.4%) and perceive usefulness of antibiotics in treating common cold and flu (77.5%).

### 3.3. Characteristics Associated with Latent Class Profiles

Table 3 presents characteristics that are associated with being an “antibiotic avoider” and “antibiotic seeker”, with reference to being an “antibiotic appropriate”. Those of non-Chinese ethnicity (Model 2: AOR 0.71, 95% CI 0.51–0.98, *p* = 0.039) and those with lower education level (Model 2: AOR 0.60, 95% CI 0.40–0.90, *p* = 0.014) were less likely to be “antibiotic avoiders” than “antibiotic appropriates”. Even though the interaction term between age and overall trust in doctors was significant in the final model (Model 2: AOR 1.83, 95% CI 1.01–3.30, *p* = 0.045), the specific pairwise comparisons between age groups within trust levels did not reach statistical significance (Table 4). The borderline *p*-value of the interaction term and the absence of significant pairwise comparisons suggest that this finding should be interpreted with caution.

On the other hand, as shown in Table 3, males (Model 2: AOR 1.49, 95% CI 1.17–1.90, *p* = 0.001) and those of non-Chinese ethnicity (Model 2: AOR 2.12, 95% CI 1.51–2.99, *p* < 0.001) were more likely to be “antibiotic seekers” than “antibiotic appropriates”. In particular, individuals with poor knowledge of antibiotic use (Model 2: AOR 3.71, 95% CI 2.89–4.76, *p* < 0.001) and AMR (Model 2: AOR 3.51, 95% CI 1.05–11.76, *p* = 0.042), those with low eHealth literacy (Model 2: AOR 1.34, 95% CI 1.02–1.77, *p* = 0.038) and high overall trust in doctors (Model 2: AOR 2.14, 95% CI 1.44–3.17, *p* < 0.001) were more likely to be “antibiotic seekers” as well. Additionally, compared to older adults aged ≥50 years, younger adults aged 21–34 years (Model 2: AOR 3.23, 95% CI 1.92–5.43, *p* < 0.001) had a significantly higher likelihood of being “antibiotic seekers”.

The effect of age on antibiotic seeking behaviour was modified by education level. Stratified analyses showed that amongst lower educated individuals, the likelihood of being an “antibiotic seeker” increased with age (AOR 1.44, 95% CI 0.83–2.49, *p* = 0.198 for those aged 21–34 years; AOR 2.77, 95% CI 1.61–4.78, *p* < 0.001 for those aged 35–49 years; AOR 4.09, 95% CI 2.39–7.00, *p* < 0.001 for those aged ≥50 years) (Table 5).

### 3.4. Attitudes Towards Antibiotic Resistance and Public Communication Channels for AMR Education Across Antibiotic Behavioural Profiles

**Figure 2:**
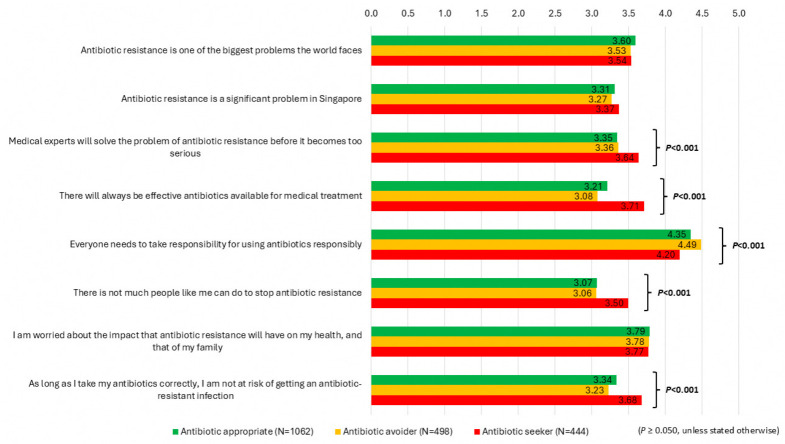
Mean scores of statements on attitudes towards antibiotic resistance across latent class profiles for antibiotic use behaviours (N = 2004).

The three antibiotic behavioural profiles did not differ significantly in their agreement that antibiotic resistance is a major problem (both globally and in Singapore) or in their concerns about the impact of AMR on their health and that of their families (Figure 2). However, “antibiotic seekers” displayed distinctive misperceptions and poor attitudes towards AMR. They trusted medical experts to solve the problem of AMR and were confident that effective antibiotics would remain available for medical treatment. Whilst they believed that correct antibiotic use practices would protect them from AMR, they were reluctant to accept personal responsibility for responsible use of antibiotics and were most pessimistic about their personal ability to stop antibiotic resistance.

**Figure 3:**
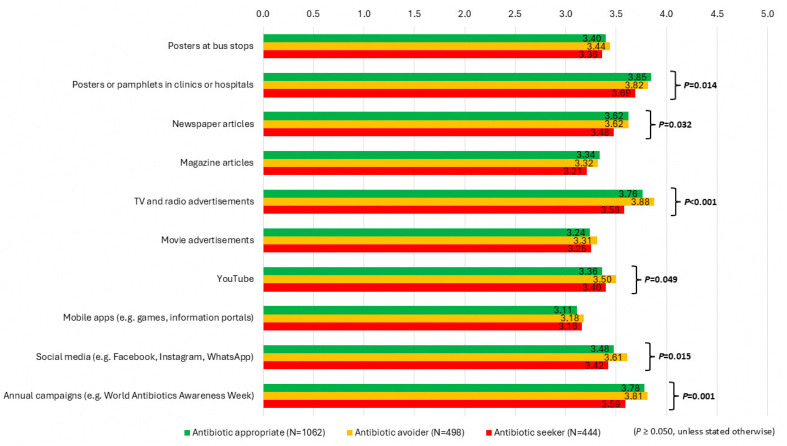
Mean scores perceived effectiveness of public communication channels for AMR education across latent class profiles for antibiotic use behaviours (N = 2004).

As for AMR education, “antibiotic seekers” were least likely to perceive that posters or pamphlets placed in clinics or hospitals, newspaper articles, TV and radio advertisements, social media, and annual campaigns are effective channels of communication (Figure 3).

## 4. Discussion

Our findings revealed three distinct antibiotic behavioural profiles in Singapore, confirming the multidimensional nature of antibiotic behaviours beyond traditional categorical classifications. Nearly three-quarters of Singapore residents did not demonstrate clearly inappropriate antibiotic practices, comprising “antibiotic appropriates” (53.0%) and “antibiotic avoiders” (24.9%). Both profiles showed low proportions of taking leftover antibiotics or stopping prescribed antibiotic courses prematurely. However, the two profiles were meaningfully distinct. Whilst “antibiotic appropriates” were characterised by restrained antibiotic expectations and limited reliance on alternative treatments, “antibiotic avoiders” were distinguished by their universal use of over-the-counter medication and rest, frequent use of complementary and alternative medicine and notably greater social influence. Such observations align with previous research demonstrating that diverse social networks help gatekeep inappropriate antibiotic use by enhancing individuals’ self-care capacity [17]. Nonetheless, the reliance of “antibiotic avoiders” on complementary and alternative medicine warrants further investigation, as the appropriateness and safety of these alternative management strategies in the context of antibiotic decision-making remain unclear.

In this study, comprehensive profiling presents a markedly different picture of inappropriate antibiotic users. While traditional categorical classifications identified 61.1% of respondents engaging in misuse behaviours [10], our profiling approach revealed only 22.2% as problematic “antibiotic seekers”. This profile extends beyond traditional indicators of seeking antibiotics from multiple doctors, using existing antibiotic stockpiles and poor adherence to prescribed antibiotic courses, to include underlying attitudes such as high expectations and perceived usefulness of antibiotics for URTI. Rather than suggesting that traditional approaches are inaccurate, this analysis highlights that latent class analysis provides an alternative and potentially more nuanced behavioural classification, capturing complex co-occurring behavioural patterns that characterise true misuse instead of treating individual indicators as absolute markers of inappropriate behaviour.

Further examination of the determinants of the “antibiotic seeker” profile showed that poor knowledge of antibiotic use and AMR, along with low education level, remained as strong attributes, not differing from what was previously identified [10]. Specifically, older adults with lower education levels are more likely to be represented in the “antibiotic seeker” profile. Furthermore, with low eHealth literacy, individuals with the “antibiotic seeker” profile would be incapable of searching and appraising health information gathered from electronic sources [18], limiting them from making informed health choices [19]. These findings not only highlighted the continued need for public education efforts but also emphasised the need for messages to be designed in lay language to accommodate individuals with limited literacy.

Compounding these information barriers, the attitudes of “antibiotic seekers” towards AMR further undermine their capacity to make appropriate antibiotic decisions. Despite recognising AMR as a significant threat, “antibiotic seekers” were reluctant to accept personal responsibility for responsible use of antibiotics and were pessimistic about their personal ability to stop antibiotic resistance. Their dependence on medical experts to solve AMR problems and misplaced confidence that effective antibiotics would continue to remain available for their future medical treatment further demonstrates a classic collective action problem where these individuals acknowledge the societal threat while avoiding personal accountability.

Patient education to address knowledge gaps and misperceptions can be delivered through primary care doctors, given the high trust that “antibiotic seekers” have in doctors in general, and particularly through regular family doctors for older adults [12]. However, this association between high overall trust in doctors and “antibiotic seeker” membership warrants a more careful interpretation. Trust in doctors amongst this subgroup may reflect deference to medical authority or expectation of receiving antibiotic prescriptions, rather than receptiveness to education, suggesting that trust alone may not be sufficient to facilitate behaviour change. While educational materials can effectively improve knowledge, attitudes and practices amongst the general public [20], conventional methods may not be adequate for this subpopulation. Their low receptiveness to traditional approaches, such as posters, pamphlets, advertisements, social media and mass campaigns, necessitates more personalised communication strategies. A unified medical record system in primary care settings would enable doctors to identify frequent doctor-hopping behaviours and deliver targeted educational interventions during consultations, creating opportunities for discussions and shared decision-making on appropriate antibiotic use [12].

The latent class analysis methodology employed in this study strengthens the literature by providing a more nuanced understanding of antibiotic use behaviours amongst the general population, an approach rarely adopted locally or internationally. This refreshed perspective sheds light on how future AMR interventions should be designed and carried out. The population-based design of this study, together with the acceptable response rate and broad demographic representativeness across most characteristics, enabled confidence in interpreting the data and reduced selection bias that could jeopardise generalisability of the findings.

However, the response rate of 41.8% raises the possibility of non-response bias, and respondents aged 50 years and above and those with lower education levels were slightly underrepresented relative to the Singapore Census 2020. As sampling weights were not incorporated into the analyses, the prevalence estimates of the identified profiles should therefore be interpreted with some caution. A key limitation of this study is that the survey items captured general dispositions towards antibiotic use rather than observed decisions of specific clinical episodes. As such, responses may reflect cumulative experiences across multiple illness episodes in the past and may not accurately represent actual behaviour in any given clinical encounter. Future research should therefore explore antibiotic use behaviours within specific clinical contexts to better understand how these dispositions translate into real-world decisions. Furthermore, the classification of AMR knowledge was strict, where a single incorrect response was sufficient to define poor AMR knowledge. This had resulted in a skewed distribution with 97.0% of the sample classified as having poor AMR knowledge. While this approach was adopted to maintain methodological consistency with our previous study [10], the limited variability in this variable may have reduced its discriminative value in the analyses. Lastly, responses may be subjected to some degree of recall and social desirability bias, though the anonymous nature of the survey likely minimised reporting bias.

## 5. Conclusions

Approximately one-in-five Singapore residents fulfil the profile of “antibiotic seekers”, with serious misperceptions that they do not need to take responsibility for using antibiotics responsibly. Their tendency to clinic hop for antibiotics suggests that primary care settings may offer opportunities for targeted educational interventions to reduce antibiotic misuse, though further research is needed to identify effective engagement strategies for this subgroup.

## Figures and Tables

**Table 1:** Basic characteristics of respondents (N = 2004).

Characteristics	N (%)
***Age group***	
21–34 years old	615 (30.7)
35–49 years old	658 (32.8)
≥50 years old	731 (36.5)
***Gender***	
Female	1050 (52.4)
Male	954 (47.6)
***Ethnicity***	
Chinese	1438 (71.8)
Non-Chinese (i.e., Malay, Indian, Others)	566 (28.2)
***Education level***	
Higher educated (i.e., Diploma and above)	1308 (65.3)
Lower educated (i.e., Post-Secondary and below)	696 (34.7)
***Ever used antibiotics before***	
Yes	1948 (97.2)
No	56 (2.8)
***Knowledge of antibiotic use***	
Good	1188 (59.3)
Poor	816 (40.7)
***Knowledge of AMR***	
Good	60 (3.0)
Poor	1944 (97.0)
***eHealth literacy***	
High	652 (32.5)
Low	1352 (67.5)
***Overall trust in doctors***	
High	684 (34.1)
Low	1320 (65.9)

**Table 2:** Latent class profiles for antibiotic use behaviours (N = 2004).

Item	Class 1: Antibiotic Appropriate (N = 1062)	Class 2: Antibiotic Avoider (N = 498)	Class 3: Antibiotic Seeker (N = 444)	*p*-Value *
***Need recognition***				
Need1: Expect antibiotics to be prescribed by doctor if suffering from common cold/flu symptoms	65 (6.1)	46 (9.2)	313 (70.5)	**<0.001**
Need2: Will take antibiotics to prevent cold/flu from getting worse	17 (1.6)	32 (6.4)	323 (72.8)	**<0.001**
***Information seeking***				
Info1: Know where to look for information on health when need advice or assistance for health-related matters like medication, diseases, health and general well-being	938 (88.3)	466 (93.6)	366 (82.4)	**<0.001**
Info2: Able to seek advice from a doctor, when need advice or assistance for health-related matters like medication, diseases, health and general well-being	1014 (95.5)	479 (96.2)	430 (96.9)	0.450
Info3: Have family members with whom feel comfortable to discuss health issues with	762 (71.8)	417 (83.7)	271 (61.0)	**<0.001**
Info4: Have friends with whom feel comfortable to discuss health issues with	656 (61.8)	367 (73.7)	247 (55.6)	**<0.001**
***Alternative evaluation***				
Alt1: When unwell, see a doctor to manage symptoms	689 (64.9)	454 (91.2)	377 (84.9)	**<0.001**
Alt2: When unwell, use Western medicine (Panadol, Decolgen, Woods Cough Syrup, etc.) to manage symptoms	309 (29.1)	498 (100.0)	165 (37.2)	**<0.001**
Alt3: When unwell, use complementary and alternative medicine (Traditional Chinese Medicine, Jamu, Ayurvedic Medicine, herbal tea, vitamin, etc.) to manage symptoms	161 (15.2)	310 (62.3)	65 (14.6)	**<0.001**
Alt4: When unwell, rest and let body recover on its own	456 (42.9)	498 (100.0)	160 (36.0)	**<0.001**
***Antibiotic obtaining***				
Obt1: See another doctor if doctor does not give antibiotics	20 (1.9)	6 (1.2)	87 (19.6)	**<0.001**
Obt2: Take leftover antibiotics based on personal judgement	37 (3.5)	25 (5.0)	164 (36.9)	**<0.001**
***Antibiotic consumption***				
Cons1: Stop taking antibiotics upon starting to feel better	196 (18.5)	112 (22.5)	295 (66.4)	**<0.001**
Cons2: Stop taking the antibiotic upon experiencing side effects	828 (78.0)	393 (78.9)	383 (86.3)	**0.001**
***Post-consumption evaluation***				
Eval1: Perceived absence of harm from taking antibiotics	144 (13.6)	102 (20.5)	228 (51.4)	**<0.001**
Eval2: Worry about the side effects of antibiotics	634 (59.7)	283 (56.8)	298 (67.1)	**0.004**
Eval3: Perceived usefulness of antibiotics in treating common cold and flu	364 (34.3)	232 (46.6)	344 (77.5)	**<0.001**

* Bolded values indicate statistical significance of <0.05.

**Table 3:** Multinomial regression analyses to identify characteristics predicting latent class profiles for antibiotic use behaviours (N = 2004).

Variables	Model 1				Model 2			
	Antibiotic Avoider vs. Antibiotic Appropriate		Antibiotic Seeker vs. Antibiotic Appropriate		Antibiotic Avoider vs. Antibiotic Appropriate		Antibiotic Seeker vs. Antibiotic Appropriate	
	AOR (95% CI)	*p*-Value *	AOR (95% CI)	*p*-Value *	AOR (95% CI)	*p*-Value *	AOR (95% CI)	*p*-Value *
***Age group***								
≥50 yo	Ref	-	Ref	-	Ref	-	Ref	-
35–49 yo	1.40 (1.06–1.85)	**0.017**	1.08 (0.79–1.47)	0.626	1.17 (0.81–1.68)	0.411	1.50 (0.87–2.58)	0.143
21–34 yo	1.71 (1.27–2.28)	**<0.001**	1.64 (1.19–2.24)	**0.002**	1.34 (0.92–1.97)	0.128	3.23 (1.92–5.43)	**<0.001**
***Gender***								
Male	0.96 (0.77–1.19)	0.695	1.44 (1.13–1.83)	**0.003**	0.95 (0.76–1.18)	0.628	1.49 (1.17–1.90)	**0.001**
***Ethnicity***								
Non-Chinese	0.80 (0.61–1.04)	0.090	1.66 (1.29–2.14)	**<0.001**	0.71 (0.51–0.98)	**0.039**	2.12 (1.51–2.99)	**<0.001**
***Education level***								
Lower educated (Post-Secondary and below)	0.73 (0.56–0.95)	**0.022**	1.62 (1.24–2.12)	**<0.001**	0.60 (0.40–0.90)	**0.014**	3.04 (1.89–4.91)	**<0.001**
***Ever used antibiotics before***								
No	0.51 (0.21–1.25)	0.142	1.41 (0.75–2.66)	0.292	0.51 (0.21–1.24)	0.137	1.42 (0.75–2.69)	0.281
***Knowledge of antibiotic use***								
Poor	1.11 (0.88–1.40)	0.372	3.61 (2.82–4.62)	**<0.001**	1.10 (0.87–1.39)	0.424	3.71 (2.89–4.76)	**<0.001**
***Knowledge of AMR***								
Poor	1.09 (0.62–1.92)	0.768	3.63 (1.09–12.10)	**0.036**	1.10 (0.62–1.94)	0.754	3.51 (1.05–11.76)	**0.042**
***eHealth literacy***								
Low	0.79 (0.63–0.99)	**0.042**	1.38 (1.05–1.82)	**0.020**	0.81 (0.64–1.02)	0.068	1.34 (1.02–1.77)	**0.038**
***Overall trust in doctors***								
High	1.08 (0.85–1.36)	0.545	1.85 (1.45–2.37)	**<0.001**	0.73 (0.47–1.12)	0.149	2.14 (1.44–3.17)	**<0.001**
***Interaction term between age and education level***								
35–49 yo and lower educated	-	-	-	-	1.09 (0.59–2.01)	0.775	0.68 (0.35–1.32)	0.251
21–34 yo and lower educated	-	-	-	-	1.16 (0.58–2.32)	0.667	0.35 (0.18–0.71)	**0.003**
***Interaction term between ethnicity and education level***								
Non-Chinese and lower educated	-	-	-	-	1.47 (0.83–2.59)	0.182	0.63 (0.38–1.06)	0.081
***Interaction term between age and overall trust in doctors***								
35–49 yo and high overall trust in doctors	-	-	-	-	1.63 (0.90–2.95)	0.108	1.01 (0.55–1.85)	0.973
21–34 yo and high overall trust in doctors	-	-	-	-	1.83 (1.01–3.30)	**0.045**	0.63 (0.35–1.13)	0.121

* Bolded values indicate statistical significance of <0.05. AOR: Adjusted odds ratio. CI: Confidence interval.

**Table 4:** Association between overall trust in doctors and “antibiotic avoider” profile, stratified by age group.

	Antibiotic Avoider (N = 498)					
	Aged 21–34 Years (N = 178)		Aged 35–49 Years (N = 181)		Aged ≥50 Years (N = 139)	
	AOR (95% CI)	*p*-Value *	AOR (95% CI)	*p*-Value *	AOR (95% CI)	*p*-Value *
***Unadjusted***						
Low overall trust in doctors	Ref	-	Ref	-	Ref	-
High overall trust in doctors	1.33 (0.89–1.97)	0.163	1.08 (0.73–1.61)	0.700	0.69 (0.45–1.07)	0.096
***Adjusted***						
Low overall trust in doctors	Ref	-	Ref	-	Ref	-
High overall trust in doctors	1.33 (0.89–1.98)	0.164	1.18 (0.79–1.79)	0.419	0.73 (0.47–1.12)	0.149

* Bolded values indicate statistical significance of <0.05. AOR: Adjusted odds ratio. CI: Confidence interval. (reference: “antibiotic appropriate” profile).

**Table 5:** Association between education levels and “antibiotic seeker” profile, stratified by age group.

	Antibiotic Seeker (N = 444)					
	Aged 21–34 Years (N = 159)		Aged 35–49 Years (N = 120)		Aged ≥50 Years (N = 165)	
	AOR (95% CI)	*p*-Value *	AOR (95% CI)	*p*-Value *	AOR (95% CI)	*p*-Value *
***Unadjusted***						
Higher educated	Ref	-	Ref	-	Ref	-
Lower educated	1.57 (0.96–2.56)	0.069	2.39 (1.54–3.71)	**<0.001**	3.33 (2.17–5.12)	**<0.001**
***Adjusted***						
Higher educated	Ref	-	Ref	-	Ref	-
Lower educated	1.44 (0.83–2.49)	0.198	2.77 (1.61–4.78)	**<0.001**	4.09 (2.39–7.00)	**<0.001**

* Bolded values indicate statistical significance of <0.05. AOR: Adjusted odds ratio. CI: Confidence interval. (reference: “antibiotic appropriate” profile).

## Data Availability

The datasets used and/or analysed during the current study are available from the corresponding author on reasonable request.

## Supplementary Materials

The following supporting information can be downloaded at: https://www.mdpi.com/article/10.3390/antibiotics15070671/s1, Table S1: Items measuring antibiotic use behaviours, and their respective response distribution (N = 2004); Table S2: Model selection for latent class analysis; Table S3: Item response probabilities by latent class for antibiotic use behaviours; Table S4: Model selection for multinomial logistic regression.
