# Supplementary material for: Identifying Distinct Antibiotic Behavioural Profiles in Singapore’s General Population: A Latent Class Analysis
Source: Antibiotics (Basel). 2026 Jul 9;15(7):671. doi: 10.3390/antibiotics15070671 (PMC13403971; doi:10.3390/antibiotics15070671)
Supplement: Supplementary file 1 [file antibiotics-15-00671-s001.zip › antibiotics-4388679-supplementary.pdf]

Supplementary Table S1: Items measuring antibiotic use behaviours, and their respective response distribution (N=2004)

Item	Yes, N (%)
Need recognition	
Before the COVID-19 pandemic, I expect antibiotics to be prescribed by my doctor if I suffer from common cold/flu symptoms	424 (21.2)
Before the COVID-19 pandemic, when I get a cold/flu, I will take antibiotics to prevent my condition from getting worse	372 (18.6)
Information seeking	
Please think about the times when you need advice or assistance for health-related matters like medication, diseases, health and general well-being. – Do you know where to look for information on health?	1770 (88.3)
Please think about the times when you need advice or assistance for health-related matters like medication, diseases, health and general well-being. – Are you able to seek advice from a doctor, when you need it?	1923 (96.0)
Are there any family members with whom you feel comfortable to discuss health issues with?	1450 (72.4)
Are there any friends with whom you feel comfortable to discuss health issues with?	1270 (63.4)
Alternative evaluation	
Before the COVID-19 pandemic, when you feel unwell, what do you normally do to manage your symptoms? – See a doctor	1520 (75.9)
Before the COVID-19 pandemic, when you feel unwell, what do you normally do to manage your symptoms? – Use western medicine (Panadol, Decolgen, Woods Cough Syrup etc.)	972 (48.5)
Before the COVID-19 pandemic, when you feel unwell, what do you normally do to manage your symptoms? – Use complementary and alternative medicine (Traditional Chinese Medicine, Jamu, Ayurvedic Medicine, herbal tea, vitamin etc.)	536 (26.8)
Before the COVID-19 pandemic, when you feel unwell, what do you normally do to manage your symptoms? – Rest and let my body recover on its own	1114 (55.6)
Antibiotic obtaining	
I will see another doctor if my doctor does not give me antibiotics	113 (5.6)
I will take leftover antibiotics when I think I need them	226 (11.3)
Antibiotic consumption	
I normally stop taking antibiotics when I start feeling better	603 (30.1)
I will stop taking the antibiotic if I experience side effects	1604 (80.0)
Post-consumption evaluation	
I think there is no harm in taking antibiotics	474 (23.7)
I worry about the side effects of antibiotics	1215 (60.6)
Do you think that these conditions can be treated with antibiotics? – Common cold and flu	940 (46.9)

Supplementary Table S2: Model selection for latent class analysis

Model	AIC	BIC
2-class model	34235.15	34431.25
3-class model	33855.93	34152.89
4-class model	Failed to converge	

Supplementary Table S3: Item response probabilities by latent class for antibiotic use behaviours

Item	Class 1 (54.4%)	Class 2 (22.3%)	Class 3 (23.3%)
Need recognition			
Expect antibiotics to be prescribed by doctor if suffering from common cold/flu symptoms	6.1%	9.7%	67.2%
Will take antibiotics to prevent cold/flu from getting worse	2.3%	8.2%	66.4%
Information seeking			
Know where to look for information on health when I need advice or assistance for health-related matters like medication, diseases, health and general well-being	95.7%	96.4%	96.0%
Able to seek advice from a doctor, when I need advice or assistance for health-related matters like medication, diseases, health and general well-being	88.7%	93.0%	83.1%
Have family members with whom feel comfortable to discuss health issues with	72.8%	82.0%	62.1%
Have friends with whom feel comfortable to discuss health issues with	63.2%	71.8%	55.9%
Alternative evaluation			
When unwell, see a doctor to manage symptoms	66.6%	89.4%	84.5%
When unwell, use western medicine (Panadol, Decolgen, Woods Cough Syrup etc.) to manage symptoms	32.4%	99.9%	36.9%
When unwell, use complementary and alternative medicine (Traditional Chinese Medicine, Jamu, Ayurvedic Medicine, herbal tea, vitamin etc.) to manage symptoms	16.8%	63.9%	14.4%
When unwell, rest and let body recover on its own	46.4%	98.0%	36.4%
Antibiotic obtaining			
See another doctor if doctor does not give antibiotics	1.7%	1.6%	18.6%
Take leftover antibiotics based on personal judgment	3.4%	5.4%	35.2%
Antibiotic consumption			
Stop taking antibiotics upon starting to feel better	18.6%	22.4%	64.3%
Stop taking the antibiotic upon experiencing side effects	77.9%	79.1%	85.9%
Post-consumption evaluation			
Perceived absence of harm from taking antibiotics	14.2%	20.3%	49.0%
Worry about the side effects of antibiotics	59.6%	56.8%	66.6%
Perceived usefulness of antibiotics in treating common cold and flu	34.6%	46.4%	76.0%

Supplementary Table S4: Model selection for multinomial logistic regression

Model #	Variables	AIC	BIC	*P*-value
1	Basic demographics [age, gender, ethnicity]	3973.664	4029.693	-
2	Basic demographics + education level	3924.335	3991.570	<0.001
3	Basic demographics + education level + ever used antibiotics before	3921.523	3999.963	0.033
4	Basic demographics + education level + ever used antibiotics before + knowledge of antibiotic use	3813.910	3903.556	<0.001
5	Basic demographics + education level + ever used antibiotics before + knowledge of antibiotic use + knowledge of AMR	3811.072	3911.924	0.033
6	Basic demographics + education level + ever used antibiotics before + knowledge of antibiotic use + knowledge of AMR + eHealth literacy	3802.633	3914.691	0.002
7	Basic demographics + education level + ever used antibiotics before + knowledge of antibiotic use + knowledge of AMR + eHealth literacy + overall trust in doctors	3781.963	3905.227	<0.001
8	Basic demographics + education level + ever used antibiotics before + knowledge of antibiotic use + knowledge of AMR + eHealth literacy + overall trust in doctors + age*education level	3775.190	3920.866	0.005
9	Basic demographics + education level + ever used antibiotics before + knowledge of antibiotic use + knowledge of AMR + eHealth literacy + overall trust in doctors + age*education level + ethnicity*education level	3772.598	3929.479	0.037
10	Basic demographics + education level + ever used antibiotics before + knowledge of antibiotic use + knowledge of AMR + eHealth literacy + overall trust in doctors + age*education level + ethnicity*education level + age*overall trust in doctors	3770.622	3949.915	0.041
